# Achieving our highest potential through character development (Husn-i-Akhlaaq): an Islamic perspective on holistic personality development, spiritual growth and human flourishing

**DOI:** 10.3389/fpsyg.2025.1569393

**Published:** 2025-08-13

**Authors:** Uzma Gillani, Muhammad Jahanzeb Khan

**Affiliations:** Department of Psychology, University of Peshawar, Peshawar, Pakistan

**Keywords:** character development, highest potential, holistic personality development, spiritual growth, human flourishing, physical health, mental health, social transformation

## Abstract

This article proposes a theoretical framework for human personality development based on Husn-i-Akhlaaq, the Islamic model of character refinement as the key to achieving one’s highest potential (Self-Actualization). Islam designates human beings as Allah’s viceregents on Earth, yet this status is not inherent; it is realized through a structured process of moral and spiritual growth. Drawing from Islamic thought, particularly Rumi’s Universal Man and Iqbal’s Mard-i-Momin, we argue that personal perfection is not just an abstract or metaphysical state but a measurable and attainable goal. This theory integrates the Ruh (Soul), Qalb (Heart), and Nafs (Self) as essential faculties in character formation, positioning Husn-i-Akhlaaq as playing a pivotal role in this holistic framework for personality development. We hypothesize that this model not only fosters spiritual excellence but also has direct implications for psychological resilience, emotional wellbeing, and overall human flourishing. By bridging Islamic teachings with contemporary psychological discourse, this article advances a structured, interdisciplinary approach to character education and character development, emphasizing its potential applications in physical health, mental health, personal growth, and social transformation.

## Introduction

Man is Allah’s viceregent on Earth, honored with the title Ashraful Makhluqaat (The Best of Creation), but he only becomes truly deserving of this status when he develops his character. This journey of character refinement is what transforms a person into the Perfect Man, as Rumi describes (Rumi, 2012), or Mard-i-Momin, as Iqbal envisions (Iqbal, 1915; Nadvi, 2015). In Islam, achieving this ideal is not an abstract notion but rather the realization of one’s highest potential, what contemporary psychology terms self-actualization, a tangible goal realized through continuous moral and spiritual effort. But, whereas in contemporary psychology, self-actualization is regarded as the realization of one’s full potential, often seen as the pinnacle of human psychological development. In Islam, this concept transcends mere psychological fulfillment, embodying a spiritual transformational journey guided by divine principles. The Perfect Man (Insan-i-Kamil) is an ideal who embodies the fullest potential of human capabilities. The Supreme viceregency, universal and comprehensive, was manifested in the Prophet Muhammad ﷺ (Al-Jillani, 2018). Following the Prophet ﷺ the Perfect Man serves as a model of how individual fulfillment aligns with universal harmony and the welfare of all creation.

The Perfect Man is distinguished by an intense love for Allah. In Islamic teachings, love for Allah is not abstract; it demands action by aligning one’s life with divine attributes, such as mercy (*rahma*), justice (*‘adl*), and compassion (*ihsan*) in one’s dealings with others. The Prophet Muhammad ﷺ is the perfect exemplar of love for Allah. The Quran describes him as “*rehmatul-lil-aalaamin”* (mercy to all worlds) (Quran 21:107), and his life is the most comprehensive model of how divine love translates into compassionate service to humanity. The Prophet’s character—*Husn-i-Akhlaaq*—provides a structured framework for character development. This framework involves conscious cultivation of virtues, avoidance of vices, fulfilling our duties and obligations, and exhibiting the best etiquettes/manners, consistent with the refinement of one’s character through self-discipline and reflection. This framework teaches that true self-actualization is not self-centered but is inherently other-oriented, living with concern for the wellbeing of others and striving for harmony and justice in the world. Thus, the journey of becoming the Perfect Man is a transformative process where divine love manifests in committed service to humanity.

Islam has put forward the most comprehensive and complete explication of character, based on its holistic approach to human personality development (Surbakti et al., 2024), in which spiritual health is as important as physical or mental health (Ali, 2024) because it is grounded in the purpose and meaning of life. In fact, in Al-Ghazali’s words, the purpose of man in this world is the development of the soul through the moral (Rizvi, 1994), which is only possible through spiritual growth and that in turn is possible through character development (Naseem, 1979; Al-Jillani, 1998; Rumi, 2012; Rumi, 1974; Bacha, as cited in Bukhari, 2005). Spirituality in Islam is nothing but the height of character (Al-Ghazali, 1938). Maslow (1968, 1971) also added a sixth need at the top of his hierarchy of needs; the need for transcendence or to expand one’s sense of meaning in life through the development of a more “spiritual” perspective. Much of Adler’s later work was devoted to the exploration of the need for transcendence because he came to believe that people need to connect with something larger than the individual self (Ansbacher and Ansbacher, 1970). Similarly, the last stage in Erikson’s psychosocial stages of development is also related to spiritual development (Erikson, 1968).

Islam regards man as the sum of a body and soul. He was given a soul because the essence of human beings is spiritual; he was given a body in the physical dimension, as it was indispensable for the expression of his being here. The body is the ride of the soul in this world (Al-Ghazali, 1981). Both have needs that must be fulfilled, to ensure the body’s health and survival on the one hand, and the soul’s health and development on the other hand. They are interdependent for their health and growth, and for the expression of one’s being as a unified whole. The Islamic conceptualization of character finds support in some other Western scholars work on character, too e.g., good character is seen in displays of high ethical standards and self-transcendence. Good character must involve the ability to “get out of” oneself, i.e., to transcend one’s personal concerns and to empathically identify with another’s feeling or situation. It is the summation of one’s virtues and a willingness to behave according to these virtues even in difficult social situations (Cloniger et al., 1993).

Allport describes his concept of “propriate functional autonomy” as the highest level of organization in human personality that determines the “total posture” of a mature life system. A prominent ingredient of this propriate organization is the sense of responsibility one takes for one’s life. He presses towards a unification of life which involves finding answers to the problems of life, relating himself to other human beings, to discover his peculiar place in the world, and to establish his identity. In all this, the sense of selfhood is involved, and selfhood reflects this fundamental human process of becoming. But, Allport says it is not easy to determine how propriate functional autonomy comes about, partly because there is a lack of sufficient knowledge of the underlying neurological processes and partly because there is yet “no consistent theory” of the nature of man (Allport, 1961). Adler’s concept of ‘social interest” (Ansbacher and Ansbacher, 1970) and Jung’s ‘unity archetype’ (Shamdasani, 2003) also drive home the importance that Islam accrues to character in human personality development, especially to its essential and highest potential.

**Figure 1:**
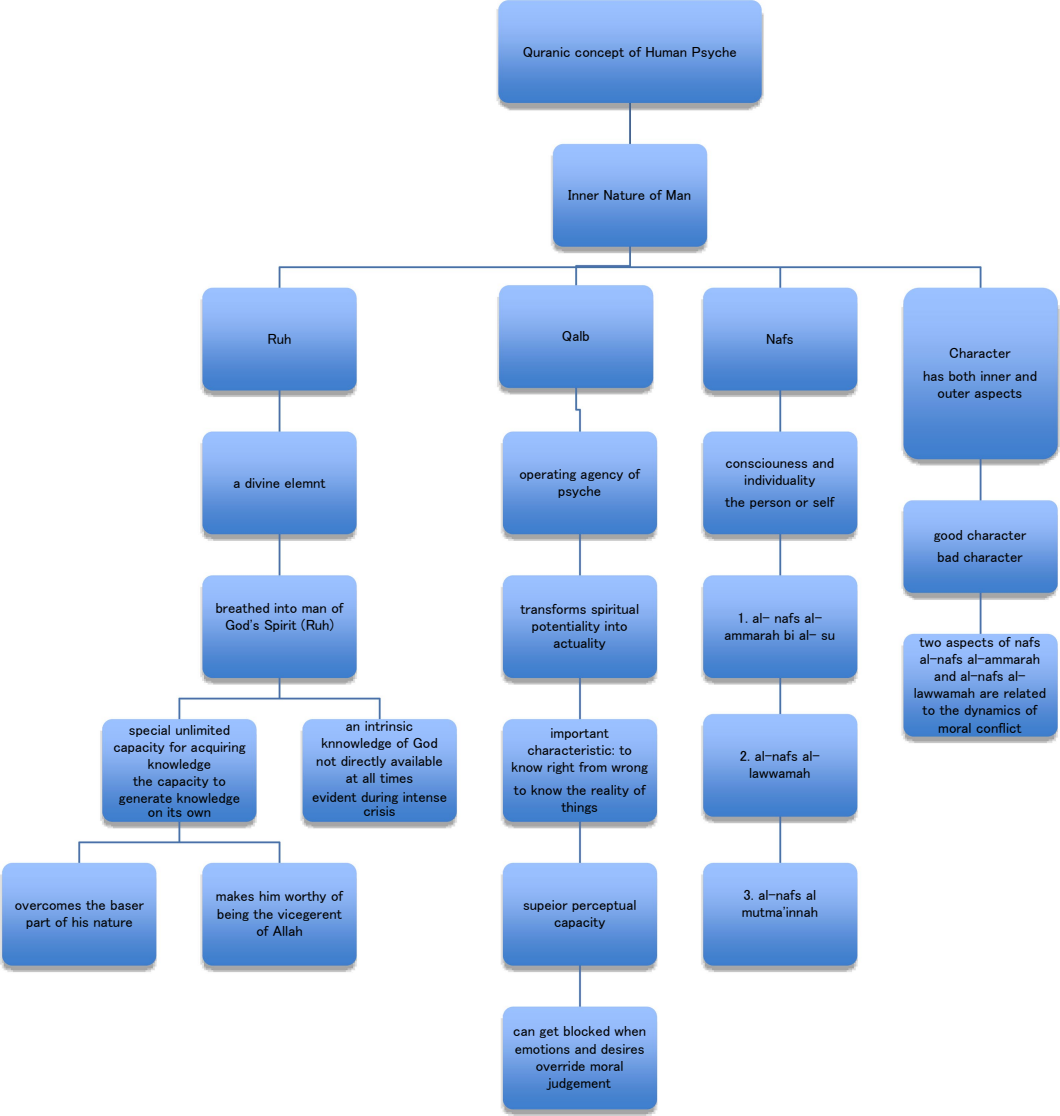
Quranic concept of human psyche.

To become the Perfect Man and achieve self-realization, he must go through a process of personality development, which involves both his body and soul and their different faculties. According to the Quran, man, as the vicegerent of God, has been created with a definite purpose. This is very different from considering man as merely another animal who came into existence as a result of a biological accident. He is also not inherently evil, who is concerned only with the gratification of immediate needs. The Quran provides valuable insight about man and his nature. The central theme of the Quranic discourse about man is his inner nature or psyche, with its social, moral, and spiritual aspects. Terms used by the Quran for it are Ruh (Soul), Qalb (Heart), and Nafs (Self) (see Figure 1), however, without mentioning character development, the picture seems incomplete. According to the theory of the Perfect Man, these are interdependent, and character development is the process that makes use of all these faculties and enables man to reach his highest potential, i.e., become the perfect man by overcoming the baser aspect of his nature and developing his higher self. Having the capacity to do bad but still choosing to do good is what elevates humans’ status higher than even angels, who are only capable of doing good and have no capacity for doing bad. This might prove to be the missing theory for “propriate functioning” that Allport talked about, also a theory that puts Adler’s concept of “social interest” in its rightful place, and a theory that explains Jung’s concept of the unity of the conscious and unconscious to its ultimate culmination, as well as Maslow’s (1971) concept of transcendence.

## Structure of personality: the components

*Ruh* (soul) is a divine element, breathed into man of God’s spirit (Quran 15: 28–29). Its characteristic is that it has the capacity for acquiring unlimited knowledge (Quran 2: 30–34; Ansari, 1992), a unique capacity to generate knowledge on its own. This endowment is inherited by the descendants of Adam (AS) as a potential capacity, varying in nature from individual to individual (Al-Jillani, 2018). This capacity, if activated, will enable him to overcome the baser part of his nature and thus make him worthy of the honor being bestowed on him by Allah, i.e., being *Ashraf-ul-Makhluqaat*, the ‘best of creation’. Only then can he know his actual relationship with the universe and his Creator and fulfil his trust, the function of viscerency that he has accepted. Without this understanding, there is a danger that the teachings of religion may remain only an external dress, to be adhered to outwardly but not adhered to inwardly. When this happens, the practice of religion turns into a rule of customs and conventions, and the presence of Allah within the heart is not realized (Al-Jillani, 2018). Additionally, one other characteristic of Ruh is that it has intrinsic knowledge of God (Quran 7: 172), but this God consciousness is not readily available at all times. It only becomes evident during intense crisis (Quran 6: 63), as it is a common observation that at such times, even those people who do not believe in Allah, cry out to Him for help (Ansari, 1992).

*Qalb* (heart) is presumably the operating agency of the psyche, which transforms spiritual potentiality into actuality, as it is the only organ in the body that can communicate with the Ruh and receive unlimited true knowledge from it. An important characteristic of the Qalb is that it has the capacity to know right from wrong, to understand the reality of things, and to make evaluative judgements. It has superior perceptual capacities, which the Quran has frequently described as an extension and superior or deeper level functioning of what is being done at the surface level by the sensory organs. However, this function of the Qalb (Heart) gets blocked when emotions and desires override moral judgements. Perceptual processes of seeing and hearing are reduced to mere sensations. They become stimulations without meaning (Ansari, 1992) because the Qalb is blocked or sealed. People in such a condition the Quran says, “Have eyes with which they do not see, ears with which they do not hear” (Quran 7: 17). This process has been described at several places in the Quran for example (Quran 2: 6–7, 7: 10, 4: 15, 63: 3, and 16: 106–108).

N*afs* (Self), like Qalb and Ruh, is another word used by the Quran to indicate an important aspect of the human psyche. At the lowest level, it is consciousness and individuality, the person or self. It has three expressions:

- al- nafs al-ammarah bi al-su; the nafs impelling towards evil
- al-nafs al-lawwaamah; the blaming self
- al-nafs al-mutma’inna; the nafs at peace.

The first two aspects of Nafs are of particular interest to a student of human psyche as they are related to the dynamics of moral conflict. Al-Nafs al-Ammarah bi al-su, which impels a person to immediate gratification irrespective of moral consequences, and al-Nafs al-Lawwamah, which impels an examination of the moral aspects of any action. The main effect of al-Nafs al-Ammarah bi al-su is to paralyze the cognitive processes, and a person becomes negligent and misled (Quran 7: 179). Their behavior is characterized by thoughtlessness and immorality. On the other hand, al-Nafs al-Lawwaamah is vigilant, constantly examining and scrutinizing its actions, fighting against its selfish and immoral desires. This constant contention is the first stage of al-Nafs al-Mutma’inna, which is a state of the Nafs at peace. It is so called because in the long run, the constant struggle with the immoral part of one’s nature aroused primarily by al-Nafs al Ammarah bi al-su, is now behind oneself (Ansari, 1992). This state of Nafs is referred to at only one place in the Quran (Quran 89: 27–30) and has been attained by many through disciplining the baser part of their nature through character development.

## *Husn-i-Akhlaaq*: character in Islam

The term Husn-i-Akhlaaq, meaning “good character,” is deeply rooted in Islamic thought and has been a central concept in Islamic ethics since the early centuries of Islam. It can be traced back to the Quran’s description of the Prophet Muhammad ﷺ as possessing “an exalted standard of character” (Quran 68: 4). This verse is often cited in Islamic ethics as a divine validation of the Prophet Muhammad’s ﷺ unparalleled character, laying the foundation for the concept of Husn-i-Akhlaaq in Islam. The ethical principles derived from it offer comprehensive guidance on how to live a just, compassionate, and honorable life. The scope of it is broad, encompassing personal behavior, social relationships, economic dealings, and even governance. Highlighting its relevance in fostering moral and just societies, as it contributes to the global dialogue on universal values and ethical practices (Bhat and Nabi, 2024). Ibn Hazm’s study *Kitab al-Akhlaq wa-al-Siyar* offers insights into early Islamic perspectives on ethics (Zakariya, 2020). Al-Farabi defined ilm al-akhlaaq as a science that studies the state of the human soul, linking inner dispositions to virtuous actions. Ibn Miskawayh later expanded on this, describing akhlaaq as a stable trait of the soul that drives action without conscious deliberation (Omar, 2016). His systematization of ethics influenced subsequent Islamic philosophers, including Al-Ghazali. Al-Ghazali and other Muslim scholars made use of Greek wisdom too, but in the context of Islamic religious practice, as they defined the “Golden Mean” as the straight path of the Quran (Wade, 2010), modeled by Prophet Muhammad ﷺ during his lifetime. While many Muslim scholars have produced extant studies explaining Husn-i-Akhlaaq, they have certain shortcomings when it comes to conducting a scientific analysis of the same. For instance, (Al-Ghazali, 1938) and Miskawyah’s research, though organized, are at best philosophical. Hashmi (2006), Mubarakpuri (1995), and many other Muslim scholars delineated character traits, but their character trait lists are neither exhaustive nor organized. According to Sav-Harvi (1976), character traits are voluntary and, like habits, require motivation to be cultivated. Like Sav-Harvi, Maulana Ashraf Ali Thanvi (Rizvi, 1994) also believes in the voluntary nature of character traits; however, some of them may be dispositional also, which again are superior qualitatively if they are done intentionally.

Following the later, the most comprehensive and organized approach to Husn-i-Akhlaaq, based on the Quran and authentic sources of Hadith, was put forward by Nadvi and Naumani (1976). They delineate a multidimensional hierarchical model of character that very comprehensively captures its nomological network (Cronbach and Meehl, 1955). Thus, Husn-i-Akhlaaq’s nomological network consists of many specific character traits that are organized under four broad domains or higher-order character traits. The four broad domains are Huqooq-au-Faraiz (Obligations and Duties), Fazail (Virtues), Razail (Vices), and Aadaab (Etiquettes/Good manners), each having many specific character traits subsumed under it. Also, it has both an inner aspect, Fazail (Virtues), and Razail (Vices), and an outer aspect, Huqooq-au-faraiz (Obligations and Duties) and Aadaab (Etiquettes/Manners), and it can be good or bad; Huqooq-au-faraiz, Fazail, and Aadaab domains comprise positive character traits, and the Razail domain comprises negative character traits. Its character traits list comprehensiveness, many specific character traits under the four main domains, makes it more amenable to scientific enquiry and practical application in our day-to-day life. This is supported by the current psychological discourse on character. The most pragmatic psychological approach to character is in line with personality psychology, and specifically that of trait theory. The new psychology of traits, while recognizing individual differences as stable and general, is also cognizant of their being shaped by individual settings and also capable of change. Thus, on the one hand, while situations affect the expression or inhibition of character traits (Peterson and Seligman, 2004), on the other hand, they are necessarily voluntary and reflect choice and will (Compton, 2005; Sav-Harvi, 1976; Nadvi and Naumani, 1976).

Let us now briefly consider the model of Husn-i-Akhlaaq as explained by Nadvi and Naumani (1976), because a full detail is beyond the scope of this paper. For details, the author’s PhD thesis (Gillani, 2019) is a good source to start with. The multitude of specific character traits and the four broad domains under which they are organized are as follows.

*Huqooq-au-faraiz* (Obligations and Duties) are defined as one’s duties and obligations towards oneself, other people, animals, and even non-living things. Since, in this universe, man gets benefit from his various relationships, therefore, this demands of him that he strive for their protection, development, and proper use, and not to misuse or abuse them. His duties and obligations become others’ rights on him, which he should fulfill to the best of his ability. The list of obligations and duties is prioritized firstly and primarily according to the closeness of the relation, and secondly according to their rightful situational justification. The specific character traits (facets) grouped under the domain of Huqqoq-au-Faraiz in order of priority are as follows:

Thus, the Huqooq-au-Faraiz (Obligations and duties) domain consist of facets like; Walidain-ky*-*Huqooq (Parents’ rights), Aulaad-ky-Huqooq (Children’s’ rights), Huqooq-i-Zojain (Spouses’ rights), Ahl-i-Qarabat-ky-Huqooq (Family members’ rights), Humsayon-ky-Huqooq (Neighbors’ rights), Yateemon-ky-saath husn-i-salook (Orphans’ rights), Hajatmundon-ky-Huqooq (Needy persons’ rights), Baiwa-ky-saath husni-i-salook (Widows’ rights), Bimaaron-ky-Haqooq (Rights of the sick), Mehmanon-ky-Huqooq (Guests’ rights), Ghulaamon-ky-Huqooq (Servants’ rights), Musalmanu-ky-bahmi-Huqooq (Muslims’ mutual rights), Insaani-Baradari-ka-Huq (Human rights), and Janwaron-ky-Huqooq (Animals’ rights, including rights of plants and non-living things).

### *Fazail* (virtues)

Virtues are defined as those excellences in human character that lead to moral perfection, that are pleasing to Allah (Subhanahu-Wataala), and are beneficial for other human beings. There is accumulating scientific evidence that considers them good for both the individual and the society at large (Jankowski et al., 2020). Many virtues have been described both in the Quran and precedents of the Prophet Muhammad ﷺ, but in many a Muslim scholar’s discourses on ethics, they are neither organized nor set in order of priority (Nadvi and Naumani, 1976). The list of these virtues in order of their importance is as follows: the first five virtues are overarching, as they are indispensable for the proper execution/development, and eradication or control of all other character traits, whether positive or negative, respectively.

The Fazail (Virtues) domain comprises the five overarching facets: Ikhlaas (Sincere intention), Taqwa (Fear of Allah, an inner sense compelling us to do good and prevent us from doing bad), Tawaqul (Dependence on Allah), Sabr (Patience), Shukr (Gratitude). Then in order of priority comes Sidq (Truthfulness), Sakhawut (Generosity), Iffat-au-Pakbazi (Temperance/Chastity), Dyanatdari-aur-Amanut (Trustworthiness and Honesty), Sharm-au-Haya (Modesty), Rehum (Kindness), Adl-au-Insaaf (Justice), Ahad-ki-pabandi (Keeping promises), Ihsaan (Delivering goodness beyond other’s rights), Afu-au-Darguzar (Forgiveness), Hilm-au-Burdbari (Going soft on others), Rifq-au-Lutf (Gentleness), Tawazu-au-Khaksari (Humility and humbleness), Khush-kalami (Good speech), Eesaaar (Altruism), Aitidaal-aur-Miana-Rawi (Moderation), Khuddari-aur-Izzat-i-Nafs (Dignity and self-respect), Shujaat-au-Bahadri (Valour and Bravery), Istiqamat (Steadfastness), Huq-Goi (Saying the right/just thing), and Istighna (Un-dependence).

### *Razail* (vices)

Razail are those specific character traits that are the opposite of Fazail and inherently bad, which one should refrain from. Vices are defined in Islam as those bad character traits which Allah (Sunhanahu-Wataala) does not like, and which are forbidden by Him. Their badness is inherent, understood, and accepted as such by any intelligent person. They are forbidden because they undermine a person’s physical, mental, and spiritual wellbeing. Because of them, people face material and spiritual losses, both at a personal level and at the collective or societal level. When they become widespread among people or nations, they destroy them because they lead to many societal ills that are detrimental to people, the environment, and other species. They create hurdles in their worldly achievements and close the doors to reaching their highest potential, i.e., becoming perfect men, leading the good life, and attaining Sadaa (ultimate happiness).

Thus, the Razail (Vices) domain consist of facets like; Jhoot (Lying), Wada-Khilafi (Not keeping promises), Khayanut-aur-buddiyaanati (Untrustworthiness), Ghaddari-aur-Daghabazi (Treason and treachery), Buhtaan (Slander), Chughal-Khuri (Telling on others), Gheebut (Backbiting), Du-rukha pun (Double faced), Budgumani (Attributing ill intentions towards others), Maadaahi-aur-khushamud (Flattery), Bukhul (Stinginess), Hirs-au-Tamah (Greed and Avarice), Chori (Stealing), Rishwat (Bribery), Sood-khuri (Interest), Bughz-au-Keena (Hatred and Malice), Ghaiz-au-Ghazab (Anger), Sharab-Khuri (Drinking), Fakhar-au-Ghuroorr (Arrogance and Pride), Zulum (Cruelty), Fuhush-Goi (Bad Talk/dirty language), Riya (Showing-off), Khud-beenee-aur-Khudnumai (Self-love and self-projection), and Hasad (Jealousy).

### *Aadaab* (etiquettes/good manners)

This domain comprises facets that are defined as the essential best practices of a civilized life, exercised in different spheres of daily living. They include the most refined and beautiful etiquettes and manners, whether they relate to food, cleanliness, or even greetings (Nadvi and Naumani, 1976). Etiquettes and good manners are the necessities of a civilized life. Adherence to them makes our collective and social life more pleasant and helps individuals appear more civilized and dignified. The basic principle underlying these etiquettes and manners is that one exhibits them with such beauty and precision that they maximally benefit other people and that they are not performed in such a way that they result in others’ displeasure and discomfort.

The Aadaab (Etiquettes/good manners) domain comprises of specific character traits (facets) like; Fitri-Aadaab (Natural etiquettes), Taharat-ky-Aadaab (Etiquettes of cleanliness), Khany-ky-Aadaab (Etiquettes of eating), Aadaab-i-Majlis (Etiquettes of gatherings), Aadaab-i-Mulaaqaat (Etiquettes regarding meetings), Aadaab-i-Guftagoo (Etiquettes of conversation), Aadaab-i-Safar (Etiquettes regarding travelling), Aadaab-i-Khwaab (Etiquettes regarding sleep), Aadaab-i-Libaas (Etiquettes regarding clothes), Aadaab-i-Musarat (Etiquettes regarding happy occasions), Aadaab-i-Maatum (Etiquettes of mourning), and Mutafaraq Aadaab (Varied etiquettes/manners).

The model explained above has been empirically validated, and an empirical character assessment measure, “The Husn-i-Akhlaaq Inventory (HAI)” (Gillani, 2019), measuring the character traits mentioned above, has also been developed. It is an empirical multiaxial character assessment instrument, grounded in the perfect man theory, that fills an important scientific gap. Exploratory Factor Analysis (EFA) determined the most viable factor structure underlying Husn-i-Akhlaaq’s construct, consisting of four broad domains or higher order factors, i.e., *Huqooq-au-Faraiz* (Obligations and Duties), *Fazail* (Virtues), *Razail* (Vices), and *Aadaab* (Etiquettes/Good manners), each having 12, 22, 23, and 11 facets or lower order factors, i.e., specific character traits, respectively. First-order and second-order Confirmatory Factor Analysis confirmed this hierarchically nested measurement model, both at the lower-order or facets level, aiming at specification, as well as at the higher-order or domains level, aiming for integration of the lower-order elements (facets) into a comprehensive theoretical explanation of Husn-i-Akhlaaq (Gillani, 2019).

There is an essential and dynamic interrelationship between these broad domains of character, a positive relationship between Huqooq-au-faraiz, Fazail, and Aadaab domains, and a negative relationship between these three domains and the Razail domain (Al-Ghazali, 1981; Winters, 2012). This relationship has been empirically determined in the above-mentioned research (Gillani, 2019). It was found that the correlation between the Razail and Huqooq domain scales (r = −0.21, *p* < 0.01), between Razail and Fazail domain scales (r = −0.63, *p* < 0.01), and between Razail and Aadaab domain scales (r = −0.46, *p* < 0.01) were all negative and significant. The Razail domain scale thus provides discriminant validity evidence for the other three domain scales. While the positive character traits scales provide convergent evidence for each other. Thus, the Huqooq and Fazail domain scales (r = 0.46, *p* < 0.01), the Huqooq and Aadaab domain scales (r = 0.30, *p* < 0.01), and the Aadaab and Fazail domain scales (r = 0.73, *p* < 0.01) showed significant positive correlations with each other.

According to Al-Ghazali, Good character, i.e., Husn-i-Akhlaaq, is, in truth, the beauty of the soul and, like the beauty of the body, it depends on the harmonious and proportionate development of all its elements (Rizvi, 1994). In Psychology and Philosophy, good character traits (positive character traits) are called virtues, and bad character traits (negative character traits) are called vices (Rizvi, 1994; Nadvi and Naumani, 1976; Garrett, 2005). The former, Islam encourages to be adopted and the latter to be removed from oneself or controlled. For, if one is satisfied simply with the removal of bad qualities, he is like a farmer who, after ploughing the field and cleaning it of weeds, sits down in expectation of the harvest, without having sown anything in the field at all. And if one only develops good character traits and does not weed out bad character traits, it is like planting a healthy seed in a diseased soil, thus thwarting or jeopardizing its growth altogether (Al-Ghazali, 1981).

Islamic character is mostly other-oriented; the welfare of others and society is at its heart, i.e., it is mostly related to huqooq-ul-ibaad or the rights of others. But learnt and exhibited purely to gain Allah’s pleasure and needs to be in alignment with one’s intuition, logic, ethics, genuine happiness, and social responsibility. The extent of this alignment determines one’s spiritual development, with more alignment indicative of a higher spiritual rank (Nadvi and Naumani, 1976). Islam emphasizes huqooq-ul-ibad (rights of others) more than huqooq-ul-Allah (rights of Allah) because serving humanity is an expression of loving Allah and following the Prophet ﷺ. While for the individual, the development of good character entails the progress of the soul and the realization of his full potential, for the society, it ensures smooth running and tranquility. Eventually, for the world at large, it means happiness and peace. In Islam, character spans all our habits that cut across all our social interactions, transactions, and relationships with others (Hashmi, 2006). Therefore, Islam provides complete details regarding the various situations and conditions that call for the expression or repression of these character traits. Unlike some recent psychologists and the Greek philosophers who made the attainment of happiness the goal of the “good life,” in Islam, both happiness, called “Sada” (Sav-Harvi, 1976), and the ‘good life’ are the natural consequence of Husn-i-Akhlaaq. Something aspired to in some most recent psychology discourses also (Ruggeri et al., 2020).

### The comprehensiveness of Islamic teachings on character

Just like the best governance is the most detailed one, which maximally eradicates bad practices by comprehensively addressing its various aspects. In the same way, the best character education is one that gives the most detail of its positive and negative aspects, explicitly enumerating every aspect of it, so that no chances of misunderstanding are left for the common man (Nadvi and Naumani, 1976). Given the human tendency to justify and find excuses for their unethical behavior or absence of ethical behavior, it will not suffice to provide them with only general principles regarding character. Taking refuge in verbosity, they would think it sufficient to provide lip service to just a few rituals. Therefore, the details of positive, negative, and neutral character traits should be made explicit along with their situational themes (Peterson and Seligman, 2004) or ecologies, as Bronfenbrenner put it (Bronfenbrenner, 1986). The key to understanding the concept of character and ensuring its application in real life does not benefit from abstraction but from explaining real-life details regarding it. This is of utmost importance in the Islamic conceptualization of character, in which both character education and acting on this knowledge in our real life are of utmost importance. This point has been accorded due consideration in the Prophet Muhammad’s ﷺ teachings on character, as evidenced by the details of various character traits making up the Husn-i-Akhlaaq concept given above. The predominance and distinction of Islamic teachings on character, i.e., Husn-i-Akhlaaq, is its comprehensiveness and practical appeal.

## The process

There is a dynamic, essential and ever evolving interplay between these components of human psyche (Ruh, Qalb, Nafs) on the one hand, and on the other hand the relationship between them and our behavior, both overt and covert, (comprising the full repertoire of character traits) which could be harmonious and growth enhancing or disharmonious and destructive. The Qalb (Heart’s) characteristic function to know the reality of things is because it is the only organ in the body that can communicate with the Ruh (soul) and receive unlimited true knowledge from it, but this can only happen if the heart is healthy (Ansari, 1992). The central role of the Qalb (heart) in this situation has been described in a hadith (precedent of the Prophet ﷺ) which says, “Beware! There is a piece of flesh in the body. If it is healthy, the whole body is healthy. If it becomes unhealthy, the whole body becomes unhealthy—that is heart” (Bukhari, 1985; Bukhari 1999).

A healthy heart is like a clean mirror that reflects the knowledge of the Ruh (Rumi, 2012; Rumi, 1974). A condition of the heart where it intuitively accesses the unlimited knowledge of the soul. What is meant by a healthy heart? The fact is that in this worldly life, every instance we find ourselves in a moral conflict, a conflict between one’s moral judgment demands and one’s need for immediate, unlimited, and unjustified gratification. Every time we take a moral decision, guided by the full range of character traits, our heart gets stronger, and every time we take an immoral decision, our heart gets weaker. For example, when we show positive character traits like forgiveness, gratitude, and patience, fulfill our duties and obligations, (towards humans, animals, plants and even non-living things) adopt good speech, and refrain from bad language even in difficult times, our heart becomes stronger and healthier, and the mirror of our heart gets cleaner. This is positively related to mental and physical recovery and health, at the individual, interpersonal and global level (McClintock et al., 2019)

On the other hand, immoral decisions are mostly instigated by Nafs-i-Ammara, which works on the pleasure principle and is self-centered and egoistic in nature. In this case, the mirror of our heart gets tarnished, and the knowledge we were receiving from our Ruh (Soul) becomes distorted or incomplete, since the transmission between the two is adversely affected. In other words, the information processing that is done by the heart’s inner higher-level senses is negatively affected by the display of negative character traits. For example, negative character traits like gluttony, greed, and lying, misusing and abusing humans, animals, plants and even non-living things, and/or not fulfilling our duties to other human beings, makes our heart unhealthy and undermines its function of knowing the reality of things, which has immense importance for making the right decisions in our day-to-day life. A trait, being a neuropsychic structure, has the capacity to initiate and guide behavior, both adaptive and maladaptive (Allport, 1961).

Moreover, if we continue living an unethical, immoral life, the mirror becomes completely rusty, and our contact with higher-level knowledge is lost. We make unhealthy decisions because of understanding things not as they are, but as distorted by our intellect, which, without this higher-level knowledge, is egoistic and easily dominated by our baser urges. Thus, compromising our decision-making abilities and leading us to unhealthy lifestyle practices, which not only affect our spiritual life but also our mental and physical life. The recent rise in different kinds of diseases, whether mental or physical e.g.; complex chronic diseases like hypertensions (Slepian et al., 2017), loss of interest, depression, lower psychological wellbeing (Wei et al., 2023), and cardiovascular disease (Davidson and Mostofsky, 2010) are due to such negative character traits like, lying, greed, and anger, respectively. Not to speak of the social and political injustices like the free-market society and dislocation (Polanyi, 1994), which result in addiction (Alexander, 2012). This reflects our negligence regarding our spiritual growth and development, which is dependent on a healthy heart, and the heart’s health, in turn, on our character development. This is supported by the fact that there is a character crisis on many fronts today, from the playground to the classroom, to the sports arena, to media, to business corporations, and politics (Peterson and Park, 2006).

Nafs-i-Lawwama, on the other hand, cautions us when we act on the instigations of Nafs-i-Ammara. However, the strength and effectiveness of Nafs-i-Lawwama also depend on, first, humans acquiring character knowledge and, second, acting on this knowledge, till one reaches the level of Nafs-i-mutma’inna. This transformative journey becomes sustainable and easy with the love of Allah, till a stage comes when Nafs-i-Ammara no longer instigates one to do the negative things, but helps in maintaining our true transformation by being more in tune with the moral demands of Nafs-i Lawamma. With persistent effort and training in the different character traits, it becomes more sensitive to the negative emotions created by the immoral acts, more attuned to the positive emotions created by our moral acts, and directed by a healthy heart in sync with our soul or higher self.

Allah (Subhanahu-Wataalaa) orders us to render our exterior being (body) harmonious by following the religious precepts and put our inner being in order through acquiring wisdom (Al-Jillani, 1998). Wisdom (the unlimited true knowledge of the soul) is acquired through the process of “Alchemy” as Rumi puts it. “Alchemy,” according to Rumi (Naseem, 1979), is none other than the development of character, i.e., acquiring Husn-i-Akhlaaq as taught and practiced by Prophet Muhammad ﷺ, as it enables humans to function at a higher level of performance. This is True Transformation, the subject matter of Rumi’s ‘Mathnavi’ (Rumi, 2012), Iqbal’s poetry (Iqbal, 1915), and the esoteric aspect of Islam.

**Figure 2:**
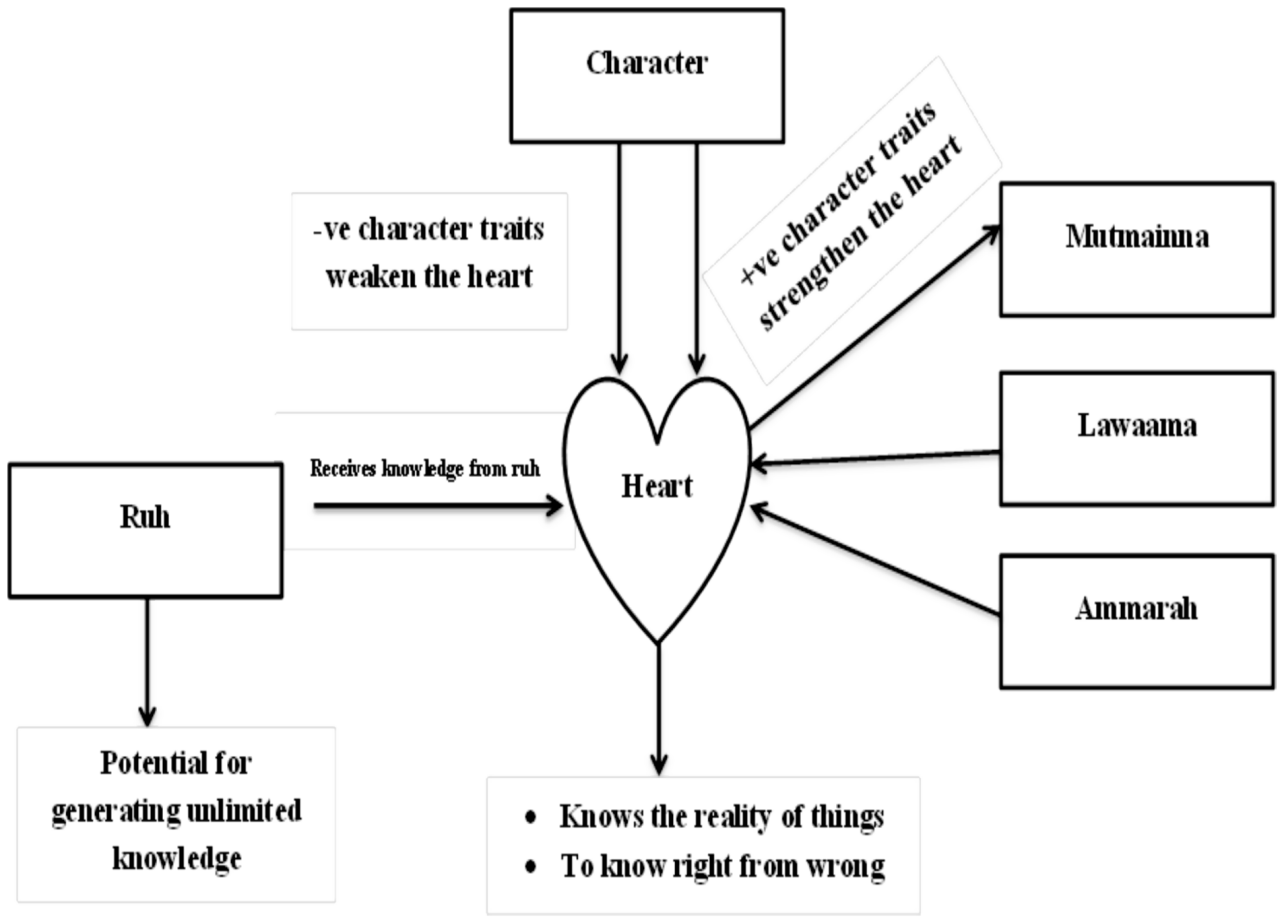
A dynamic interplay between the different components of personality.

Husn-i-Akhlaaq gives us the details of all the character traits and the relevant situational themes (Peterson and Seligman, 2004), details that delineate how best to behave in a particular situation. The more we exercise the character traits espoused by Husn-i-Akhlaaq, the more our hearts become healthier and the mirror of our hearts cleaner, thus correctly reflecting the knowledge it now receives from our higher self (Ruh/soul), a higher level of consciousness. The knowledge so gained through the purification of our hearts made possible by character development, is called wisdom (Al-Jillani, 2018; Nadvi, 2015), or the “Alixir of life” as Rumi puts it (Naseem, 1979). When the outer and the inner become one and wisdom and religion unite, one reaches the level of Truth, i.e., his ultimate potential (the perfect man), the divine purpose in the creation of man. However, proximity to the Truth is directly proportional to the amount of false materiality that one has thrown away (Ajmal, 1986) (see Figure 2).

So true, ‘Alchemy’ is not the transformation of metals into gold, but it is the ‘true transformation’ of human beings through character development, and the ‘Alixir of life’ is the acquisition of wisdom through this process (Rumi, 2012). Iqbal (1915) emphasized that intellect only guides one to one’s destination, but it is not one’s destiny. Pure intellectualism teaches one only self-centeredness, whereas for humans to reach their highest potential, i.e., to become self-actualized, they must work on the development of their hearts, as it teaches humans selflessness by being compassionate and more concerned about the welfare of others (Iqbal, 1975).

**Figure 3:**
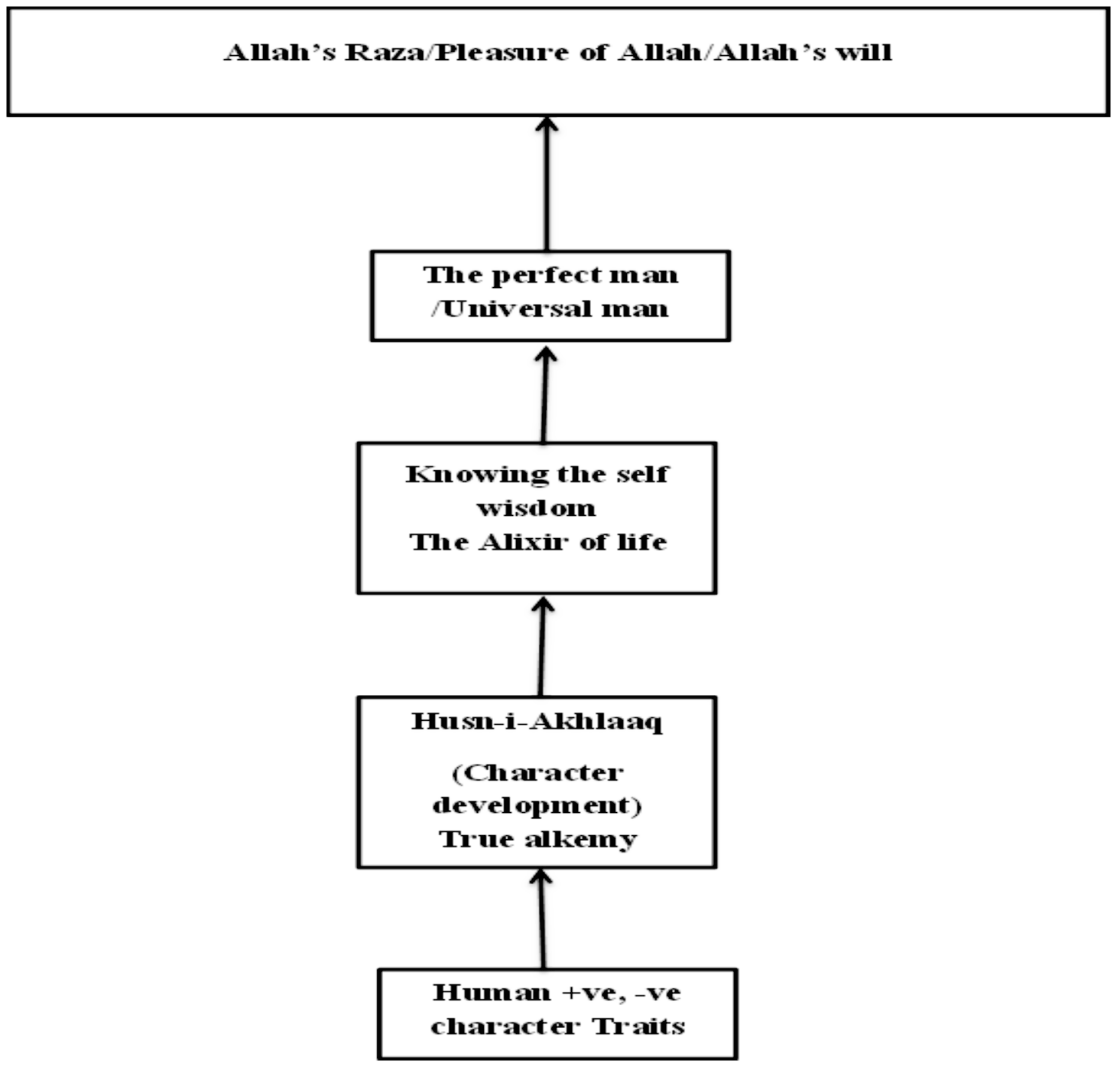
The process of becoming the perfect man.

This upward mobility of humans, reaching their highest potential (self-actualization), is only possible through the letting go of egoistic behavior. In other words, redefining the “I” as ‘what I do for others’, rather than ‘what I do for myself’. The sacrifice of animals in different religions has been a symbol of this “ego” sacrifice. But humanity, mesmerized only by the objective world, paid attention to its superficial aspect only and not to its deeper meaning, which was to sacrifice one’s ego for the greater good of others. Islam’s teachings on character called ‘Husn-i-Akhlaaq’ are other-oriented, i.e., sacrificing one’s ego for the welfare of others is the true spirit of Islam, and essential for spiritual development (Durrani, 1982, 1986) (see Figure 3).

### Scientific support

The heart is more than a physiological pump, and many cultures, religions, and spiritual traditions have regarded it as a source of intuition, love, and wisdom since antiquity (Anderson, 2020; Lind, 2015). This is the healthy heart, which has also been called the spiritual heart, intuitive heart, or energetic heart (McCraty, 2015). Just like the heart communicates with the brain through extensive afferent connections (McCraty et al., 2009), it can also connect with the higher self, i.e., with a deeper part of ourselves, referred to as the higher self or as physicist David Bohm described, “our implicate order and undivided wholeness” (Bohm and Hiley, 1993). This is our Ruh, or in scientific jargon, a higher level of consciousness.

The higher self makes a connection with the body via the healthy heart, through such unobservable processes as thoughts, emotions, and intuitions (Miller et al., 2019; Thayer and Ruiz-Padial, 2006; McCraty, et al., 2004a; McCraty et al., 2004b). These operate beyond time and space in the frequency domain. Several distinguished scientists have proposed plausible mechanisms for this interaction (Pribram, 1991; Laszlo, 2008; Schempp, 1992). This non-local intuition, the Islamic conceptualization of wisdom, is transformational as it guides our moment-to-moment experiences and interactions. Some scientists call it heart’s intelligence. Now, self-regulation of thoughts and emotions becomes more intelligent, and over time this lifts consciousness to higher levels of functioning and establishes a new, more advanced level of internal physiological and psychological baseline for performance (McCraty et al., 2004b).

This results from a stronger connection with our inner voice or our higher self. This is what is meant by knowledge reflected in the heart. This is the scientific explanation of the connection between our heart and our Ruh (Soul) or higher self. However, it is clear from this scientific evidence that only ‘a healthy heart’ can receive this higher-level awareness and intuition. As we have seen, a heart becomes healthy with character development. It is only through character development that the mind, emotions, and heart get synced, which then opens the door to higher levels of human consciousness and human functioning. Thus, enabling us to reach our highest potential (self-actualization) and become the Perfect human beings, the best of creation.

In the mid-1990s, the concept of emotional intelligence persuasively argued against the viewpoint that human intelligence is essentially mind-based. They contended that this concept of human intelligence was far too narrow. Qualities like altruism, compassion, gratitude, one’s ability to control one’s impulses (negative character traits), and self-awareness/self-realization were more important than a high IQ. These qualities, more so than IQ, enabled people to excel in the face of life challenges, and to develop heart’s intelligence or intuition. When the brain and heart, or in other words, when our mind and emotions are out of sync, it results in radical behavior changes that make us feel like two people in one body, difficulty making decisions, confusion, and a lack of alignment with our deeper core values also become evident. However, if we are mentally and emotionally synced, we feel more secure and aligned with our deepest core values and exhibit increased resilience and inner balance in the face of stressful situations. This is called heart coherence, which can lead to social coherence as well as global coherence (McCraty, 2015; Timofejeva, et al., 2017). The Global Coherence Initiative (GCI) of the HeartMath Institute (McCraty, 2015) and the Global Consciousness Project (GCP) of Princeton University (Nelson, 2010) are scientific efforts that highlight the importance of the heart’s intelligence in achieving higher levels of human consciousness at the global level.

One of the main themes of positive psychology is the good life. While so far Happiness and life satisfaction have been considered as the defining criteria of the good life (Seligman, 2002a; Honderich, 1995), future trends in this regard have started exploring other criteria for recognizing a life lived well, a life worthy of admiration and respect, a life that includes prosocial values and working towards socially desirable goals (Weziak-Bialowolska et al., 2021). Some of these other criteria include feelings of love and affection for others, realizing one’s potential, and increasing a sense of meaning by experiencing a spiritual dimension in life (Compton, 2005). Furthermore, researchers like Magnusson and Mahoney (2003) say that psychology needs to see people as more holistic, as integrated systems of mind, body, emotions (heart), and spirit.

Contemporary researchers are realizing the need for such a comprehensive theory of flourishing (Symons and VanderWeele, 2024). The Islamic concept of character, i.e., Husn-i-Akhlaaq, addresses all these criteria of the good life in its comprehensive theory of the Perfect or Universal man. The predominance and distinction of Islamic teachings on character, i.e., Husn-i-Akhlaaq, is its comprehensiveness and practical appeal. It provides both the interpersonal (between people) and intrapersonal (within a person) tools necessary for physical, mental, and spiritual health that enable people not only to overcome the many challenges of human experience but also achieve their highest potential, wellbeing, and flourishing. The former refers to the Huqooq-au-Faraiz and Aadaab domains, with a self in social context focus, and the latter to the Fazail and Razail domains with an inner integration of body, mind, and soul, along with their rightful expression or repression in a social milieu.

In this regard, the construct of Husn-i-Akhlaaq is not only comprehensive but systematic too, and it can be empirically assessed using the Husn-i-Akhlaaq Inventory (HAI) (Gillani, 2019). Thus, the 407-item HAI is a psychometrically sound measure for the assessment of character both at the lower-order or facets level, as well as at the higher-order or domains level, measuring each domain by summing scores on its facet scales. This entails that prospective users will have highly valid and reliable measures of four global domains, as well as more specific information on specific character traits within each domain. Eventually, global scores on high-order factors would become more valid when supplemented by valid facet-level scores. Grounded in a holistic theory of personality development that addresses our physical, mental, and spiritual development, this has important implications for proper assessment and precise interventions whether as wellbeing coaching programs aimed at achieving our highest potential and social and emotional wellbeing or therapeutic interventions, aimed at achieving our physical, mental, and spiritual health.

## Conclusion

This highlights the importance of character development to enable humans to reach their highest potential. As already pointed out, humans are endowed with both positive and negative character traits. The purpose of these negative character traits or baser elements of human nature was to complete his knowledge of the negative or bad too. His distinction and superiority over other species was the giving to him of complete knowledge by God (Quran 2: 30–34). Thus, in the form of Nafs-i-Ammara (the lower self), he could experience the negative character traits firsthand or indirectly through others exhibiting them. But the secret is to know them and learn to control them, rather than indulging in them. This process entails emotional suffering and requires a strong resolve to overcome it. Character education and character development help us muster this resolve and keep going on this transformative journey, for it is going through these emotional states that is conducive to man’s spiritual growth. Since the intellect, due to its reliance on the limited knowledge of the external senses, could be overwhelmed by Nafs-i-Amaara or our lower self, our ego, desires, fears, and appetites, prophets were sent to guide people in this regard, i.e., to ensure humans’ spiritual growth through character development.

If we look critically at the reasons for past nations destruction it becomes clear that it was mainly because of the negative character traits or vices they indulged in, like cheating in sales, pride, greed, cruelty, being ungrateful etc. which led to their destruction, because they missed understanding the main purpose of their creation, to become better human beings in fact to become *Aashraf ul Makhluqat ‘*the best of creation’. It therefore seems a plausible conclusion to draw that holistic personality development is not possible without the development of the soul, which in turn is only possible through an action-oriented comprehensive character development program, that then ensures our physical, metal and spiritual growth and flourishing. The heart, which is the seat of intuitive intelligence, becomes healthy during this process, and man, through heart’s intelligence, gains access to knowledge that is not possible through pure intellect. This is the definition of wisdom in Islam, and it is this intuitive knowledge that makes him the perfect man by knowing his true self, which allows man to reach his highest potential.

If a handful of positive character traits like care, love and compassion as pointed out in the HMI research can produce heart coherence and better solutions to our problems, we can well imagine what a comprehensive character development program using empirical character assessment tools like the Husn-i-Akhlaaq Inventory (Gillani, 2019; Gillani and Khan, 2021) can achieve, that encompasses the full range of specific character traits along with their minutest details, with relevance and predictive ability, for real life applications. Such positive change can result in higher levels of consciousness, ensuing human health and well-being, and greater cooperation and collaboration, among people, thus enabling us to come up with innovative solutions not only for our physical and mental diseases, but effective and sustainable solutions for our social, economic and political issues as well (Hagelin, 2007). The fact is when outdated structures, that do not serve humanity collapse, we need to replace them with more suitable and sustainable models, like the holistic personality development model discussed here, that is anchored in a comprehensive multi-axial character development process with important implications for physical and mental health, spiritual development, flourishing, social justice and global peace.

Limitations and future trends:

1. A detailed explanation of the Husn-i-Akhlaaq model especially descriptions of the specific character traits and their interrelationships could not be covered in this article, as it was more focused on explaining the holistic theory of personality from an Islamic perspective, in which the concept of Husn-i-Akhlaaq is grounded, and its implications for health, wellbeing, flourishing, and self-actualization.
2. The Husn-i-Akhlaaq Inventory (HAI) development and validation details also could not be covered here, as the test construction details warrant another article dedicated to this topic alone. The manuscript of this article is in progress.
3. A full overview of the historical background of character discourse, including the Greek philosophers, the Prophets, Islamic and Western scholarship both past and present, and the different empirical measures used to assess character traits, cannot be covered here due to the word count limit. However, they have been discussed in comparative detail and critiqued in my doctoral research.

## Data Availability

The original contributions presented in the study are included in the article/supplementary material, further inquiries can be directed to the corresponding author.
